# Exploring the Therapeutic Effects and Mechanisms of Transcranial Alternating Current Stimulation on Improving Walking Ability in Stroke Patients via Modulating Cerebellar Gamma Frequency Band—a Narrative Review

**DOI:** 10.1007/s12311-023-01632-3

**Published:** 2023-11-14

**Authors:** Tingyi Feng, Lichao Zhang, Yuwei Wu, Lin Tang, Xixi Chen, Yuanli Li, Chunlei Shan

**Affiliations:** 1 Department of Rehabilitation Medicine, Yueyang Hospital of Integrated Traditional Chinese and Western Medicine, Shanghai University of Traditional Chinese Medicine, Shanghai, China; 2 School of Rehabilitation Science, Shanghai University of Traditional Chinese Medicine, Shanghai, China; 3 Engineering Research Center of Traditional Chinese Medicine Intelligent Rehabilitation, Ministry of Education, Shanghai, China; 4 Department of Rehabilitation, Shanghai Seventh People’s Hospital, Shanghai University of Traditional Chinese Medicine, Shanghai, China; 5 Institute of Rehabilitation, Shanghai Jiao Tong University School of Medicine, Shanghai, China

**Keywords:** Stroke, Balance, Walking, Transcranial alternating current stimulation, Gamma frequency

## Abstract

The cerebellum plays an important role in maintaining balance, posture control, muscle tone, and lower limb coordination in healthy individuals and stroke patients. At the same time, the relationship between cerebellum and motor learning has been widely concerned in recent years. Due to the relatively intact structure preservation and high plasticity after supratentorial stroke, non-invasive neuromodulation targeting the cerebellum is increasingly used to treat abnormal gait in stroke patients. The gamma frequency of transcranial alternating current stimulation (tACS) is commonly used to improve motor learning. It is an essential endogenous EEG oscillation in the gamma range during the swing phase, and rhythmic movement changes in the gait cycle. However, the effect of cerebellar tACS in the gamma frequency band on balance and walking after stroke remains unknown and requires further investigation.

## Background

Muscle weakness, aberrant muscle tone, joint contractures, and other sequelae caused by a stroke can result in abnormal gait and increase the risk of falling, causing great inconvenience to the daily life activities of the patients. Normal gait is associated with good balance, postural control, adequate muscle strength, and rhythmic motor development. Additionally, patients need to have relevant motor learning skills such as environmental adaptation and information integration [1]. Clinically, most conventional rehabilitation therapies—including physical therapy and occupational therapy—are used to help patients walk more easily by improving muscle strength and muscle tone in the affected limb. However, their abnormal gait pattern is difficult to correct. This abnormal asymmetrical gait may also lead to abnormal joint loading in the lower limb, resulting in joint injury, pain, and deformity [2], forming an irregular gait pattern and raising the risk of falling. For improving abnormal gait, new techniques like neuromodulation targeting the cerebral cortex can be combined to improve the patient’s postural control and motor learning ability from the “top-down” to correct the issue. This is in addition to the traditional “bottom-up” rehabilitation therapy, which reshapes the injured cortex and subcortical network through movement and proprioceptive training of the affected limb. Non-invasive brain stimulation (NIBS) is the primary method for neuromodulation, including transcranial magnetic stimulation (TMS), transcranial direct current stimulation (tDCS), transcranial alternating current stimulation (tACS), etc., producing long-term potentiation by modulating cortical excitability. Transcranial direct current stimulation (tDCS), transcranial alternating current stimulation (tACS), and similar techniques alter the excitability of the cerebral cortex to produce long-term potentiation (LTP) or long-term depression (LTP). Long-term potentiation (LTP) or long-term depression (LTD) regulates cortical metabolism and electrophysiological activity by modulating cortical excitability [3], which has been used in treating various neurological diseases [4]. The primary motor cortex has been primarily targeted in recent studies of NIBS interventions for lower extremity dysfunction following stroke, but the effects are uneven [5–8].

Due to the extensive connection between the cerebellum and the cortex, neocortex, and inferior spinal tracts, the cerebellum can regulate posture control, balance support, and motor learning during walking [9, 10]. Additionally, studies have proved that most patients with supratentorial injuries have structurally sound cerebellum with a high level of plasticity [11–13]. Therefore, from the perspective of gait and motor learning theory, researchers have considered the research significance and viability of the cerebellum as a new target to improve gait abnormalities.

In contrast to other non-invasive brain stimulation (NIBS) techniques, transcranial alternating current stimulation (tACS) can selectively influence oscillations at the stimulated frequency through a targeted paradigm of periodic and mild perturbations. This capability allows for a more precise investigation of the mechanistic role of the cerebellum in walking and motor learning. It can aid in selecting methods for reshaping the motor brain network in stroke patients [14]. However, existing literature has only demonstrated the enhancement of lower limb function in healthy individuals with 50 Hz, specifically in the gamma frequency range, tACS, and this approach has yet to be applied to stroke patients.

Therefore, we will summarize the crucial roles of the cerebellum and gamma frequency oscillations in walking and motor learning. This will serve as a foundation to explore the application of gamma frequency cerebellar transcranial alternating current stimulation (tACS) in improving walking function in stroke patients. We conducted a comprehensive search on PubMed, Embase, and Web of Science, using keywords such as “cerebellum,” “gamma oscillations,” “tACS,” “post-stroke cortical reshaping,” “walking,” and “motor learning.” We compiled and categorized the obtained content for presentation in this review.

## Cerebellum and Gait

The cerebellum is an anatomically complex region of various structures involved in motor planning, learning, execution, and movement patterning. According to structural and functional neuroimaging studies of the neural basis of balance, the cerebellum is the most critical brain region for controlling balance, and postural control is directly correlated with cerebellar gray matter volume [15–17]. Poor balance and coordination and frozen gait can be caused by cerebellar hypoplasia or damage to the cerebellar structures [18]. The cerebellum can maintain balance by integrating somatosensory input from the head and proximal parts of the body and direct connections to the contralateral cortical motor area [5, 19, 20]. Additionally, a study in post-stroke populations has shown a direct correlation between walking ability during recovery and the degree of cerebellar activation [21]. It is clear that the cerebellum plays a crucial regulatory role in balance movements and that each of its various parts is important for postural control and motor regulation.

### Cerebellar Involvement in Balance and Postural Control in Walking

The cerebellar vermis and nodes are the vestibular cerebellum (cerebellum), and the cerebellar earth and middle portion of the hemispheres are referred to as the spinal cerebellum (old cerebellum). The combined synergy of each part plays a significant role [22] in muscle tone, posture, balance control, and limb coordination. Among them, the cerebellum is one of the supraspinal walking generators for anticipatory postural adjustment, walking initiation, and speed regulation. It is connected to the spinal motor neurons through the brainstem [23]. The sensorimotor cortex, parietal lobe, visual cortex, thalamus, basal ganglia, cerebellum, and subthalamic and midbrain (cuneate and pontine peduncle nuclei), as well as the vestibular, red, and olivary nuclei [24] that descend into the brainstem, are among the neural networks involved in walking. The cerebellum plays a crucial role in walking because of its extensive and intricate connectivity to these structures. According to the classical optimal feedback control theory [25] of postural control, the cerebellum plays a significant role in walking by being able to derive the best flexible multi-joint combinations of movement schemes and output them downward using the basic sensory integration information acquired with brain regions like the sensorimotor cortex, visual cortex, and thalamus as well as the prediction of movement sensations [26] by brain regions like the sensorimotor cortex and parietal cortex. An important location for the integration of feedback data is the vestibular cerebellum. The cerebellar nodes, the earthworm, the lobule, and the pars cerebellar are just a few of the midline and caudal cerebellum structures where vestibular protrusions from the vestibular nuclei can be found. These structures are primarily involved in integrating vestibular information to control the head and trunk position during movement. The spinal motor centers, basal ganglia, and frontal lobes [27] are also affected by the vestibular cerebellum’s projections, allowing for limb coordination and fine-tuning posture during walking through visual [28, 29] and proprioceptive feedback. The cerebellum, cortex, and spinal cord control static postural functions [30] like head and trunk tilt. This circuit can also fine-tune limb movements during walking, allowing the trunk or limbs to return to their original movement patterns and maintain dynamic balance after being perturbed by the outside world. To adapt to internal and external disturbances, this motor pattern depends on a complex interplay between the vestibular, visual, and proprioceptive systems of the spinal cord, supraspinal motor networks, and sensory feedback, in which the cerebellum plays a key role.

### Cerebellar Involvement in Motor Learning in Walking

In addition to postural control and balance coordination, the cerebellar structures’ interconnectedness can significantly affect gait in terms of its function in motor learning. First, the cerebellum can be crucial for learning new skills, and studies [31, 32] on animals have revealed that the paramedian lobule of the cerebellum’s cellular dendrites changes as new skills are learned. Lower activity in the cerebellar cortex’s lobules impacts how learned automatic tasks in the upper limbs are carried out [33]. Since “error prediction and correction” has long been thought to be the primary mechanism underlying cerebellar motor learning, olivary nucleus-cerebellar activity has been shown to enhance motor commands, and issue sustained motor commands through synaptic plasticity. The “trigger and store” theory [34] postulates that neural tracts from the olivary nucleus-cerebellar pathway connect to various cerebellum regions (like cerebellar cortical cells and nuclei). Plasticity occurs between these regions at multiple rates to correct or avoid errors, like completing classical associative motor learning, and like the blink reflex. Patients with primary cerebellar hypoplasia have been shown [35] to have impaired blink reflexes, and patients with cerebellar stroke perform significantly worse [36]on continuous finger-tapping tasks and sequential tasks in consolidation when compared to unaffected controls. Learning new cognitive operations is also influenced by the extensive cerebellar-neocortical connectivity 5 that results in cortico-cerebellar plasticity, and this motor learning also takes the form of learning new sequences [37] and predicting and correcting errors [38].

A recent study [39] demonstrates that depending on the pertinent loops and differences in signaling, motor learning patterns in the cerebellar-motor cortex, cerebellar-frontoparietal network, and cerebellar-spinal cord have complex and higher-level influences, including “prediction and measurement of movement” in addition to new skill learning and error prediction correction, and they suggest that NMDA [40] receptor-dominated LTD loops in cerebellar parallel fiber-Purkinje cells [41] (or molecular lamina) are responsible. Thus, the cerebellum can play a significant role in associative motor learning and creating and maintaining sequential movements thanks to connections between the olivary nucleus and cerebellum and the cerebellum and the neocortex. This can be observed throughout the entire gait cycle of a human. Still, it is particularly evident when walking because gait trajectories and speeds must be constantly adjusted to maintain uninterrupted walking movements to accommodate environmental constraints like shifting road and terrain conditions [42].

### Progress in the Study of the Effects of Cerebellar NIBS on Walking

Numerous NIBS interventions [43–45] targeting the cerebellum have been tested in recent years to enhance motor learning from various perspectives, proving that the cerebellum has multiple effects on motor learning via different pathways. Belkhiria C et al. [46] discovered that when faced with a route change or needing to pass through a scene with narrow terrain, activation of the cerebellar-red nucleus and cerebellar striatum was linked to motor readiness and sustained attention by functional MRI [47]. Koch G et al. [48] used TMS’s intermittent theta burst rhythm stimulation patterns to improve Berg Balance Scale scores and walking function of stroke patients. At the same time, EEG revealed specific activation of the parietal lobe, which is consistent with the combined effect of cerebellar effects on motor and frontoparietal motor learning networks to improve gait. Studies focusing on the modulation of normal and stroke gait at various targets of cerebellar NIBS in combination with traditional rehabilitation techniques like conventional physiotherapy or walking-assisted robots have grown in number [49, 50] This is due to the high plasticity of the cerebellum in movement and motor learning and the non-invasive and user-friendly benefits of NIBS. With bilateral high-accuracy cerebellar tDCS interventions in the dentate nucleus of the cerebellum and VIIb–IX in the lower extremity region of patients with posterior stage stroke, respectively, Solanki D et al. [51] demonstrated improved scores on the 10-m walk test, Time Up and Go, and Berg Balance Scale. The findings of Picelli A’s team [52, 53] lend credence to the notion that augmenting robot-assisted gait training in chronic stroke patients with cathodal tDCS stimulation of the contralateral cerebellar hemisphere and cathodal DC stimulation can be beneficial.

## Gamma Band Has a Significant Impact on Movement and Walking

EEG oscillations produced by neurons during brain activity are typically divided into five bands: beta (13–30 Hz), alpha (8–13 Hz), theta (4–8 Hz), delta (1–4 Hz), and gamma (> 30 Hz) [54]. Among them, inhibitory interneurons and pyramidal cells [55] interact to produce EEG oscillations in the gamma band. Gamma oscillations affect perception, movement, memory, and emotion differently through excitation-inhibition and inhibition-inhibition control of the specific synaptic connections between inhibitory interneurons and their peripheral excitatory neurons. Gamma rhythms are categorized as low gamma (25–40 Hz), medium gamma (40–65 Hz), and high gamma (65–85 Hz) [56] by the various options for spatial information processing in the primary visual cortex. The basal ganglia–thalamic–cortex is the primary circuit of human movement. And localized single-point transient gamma oscillations can be activated and form a gamma oscillation network [51] in multiple brain regions by coupling phase and amplitude, thus creating coherence with theta waves [57] in key brain regions such as hippocampal pus and prefrontal to influence motor learning. After a stroke, the cortical network oscillations’ excitation and inhibition are out of balance and out of sync, affecting the injured lesion locally [58] and the functional connectivity between distal cortical loops. Exogenous gamma neuromodulation has been shown to improve blood flow, motor function [59] and brain reorganization in the acute phase through cross-regional interference with spontaneous EEG oscillatory networks in cortical regions. Gamma frequency oscillations are believed to play a significant role in typical walking and the motor initiation and learning processes associated with walking.

It has been demonstrated that the gamma band oscillations in the brain itself influence gait throughout the cycle and activate various brain areas to create stable walking networks. Brain EEG detected ERD-ERS (event-related desynchronization (ERD); event-related synchronization (ERS)) lateralization [60]in the cerebellar beta-gamma frequency band during gait initiation, i.e., during leg swing and the double-support phase [61]. ERD-ERS was also found in the primary motor cortex, premotor cortex, and thalamus throughout the gait cycle. Additionally, restricted rhythmic oscillation of the upper limb during walking reduces beta-gamma ERD-ERS in the supplementary motor area (SMA) [62]. Additionally, the gait cycle and the coupling of the high and low gamma frequency bands in the sensorimotor cortex are closely related, and modifications to their coupling form affect the gait rhythm [63]. According to another study [64], the pontocerebellar pedunculopontine nucleus exhibits intrinsic oscillations in the gamma frequency band, which is crucial for subcortical rhythmic walking. Therefore, we postulate that the initiation of walking, the transition and coordination of the swing and support phases, and the maintenance of rhythm in walking are all significantly influenced by the brain’s gamma-band oscillations.

The process of regaining motor function after a stroke is broadly analogous [65] to that of motor learning, and in normal subjects, activated cortical regions, particularly the cerebellum, play a significant and influential role in the later functional recovery [66]. Rhythmic oscillations between regions of the sensorimotor cortex [67], parietal lobe, and primary motor cortex [68] can be modulated and synergized by the cerebellum, thus regulating walking-related factors such as sensory input and muscle tone during movement. It has been demonstrated that [69] the thalamus, motor-sensory cortical areas, and motor cortical areas can all be excited by gamma-band oscillations in the cerebellum via specific neuronal synaptic structures.

## Gamma-Band Cerebellar tACS for Improving Motor Function

### Overview of tACS

TACS, a noninvasive neuromodulation technique, uses weak sinusoidal electric fields to modify cortical activity. Through a more focused stimulation paradigm, tACS periodic, weak perturbations can selectively affect oscillations at the applied stimulus frequency [70]. Exogenous sinusoidal alternating current (tACS) of various frequencies directly interacts with neuronal activity sustained during rest or in the task state, impacting oscillations in particular regional brain networks by entraining, synchronizing, or interfering with them [71]. However, the underlying mechanisms by which it modifies the spatiotemporal dynamics of large-scale cortical network dynamics are still up for discussion. The effects of tACS are highly variable, with different pulse frequencies, phases, and duration of action modulated to bring the cortex into excitation-inhibition balance [72], in contrast to the NIBS approach, which produces sustained, long-duration effects by altering the excitability of the cerebral cortex, including TMS and tDCS, thereby regulating cortical metabolism and electrophysiological activity [73]and targeting the threshold of neuronal membrane potential [74].

The contraindications of tACS as a type of transcutaneous electrical stimulation (tES) can be found in the list of clinical contraindications for tES, which includes patients with epilepsy, metal in the head, pacemakers, and other implanted materials [75]. Even though tDCS and tACS fall under the same category of tES, differences in their mechanisms of action and protocol design (including current intensity and target setting) result in differences in their tolerance, discomfort, and safety. For example, the anodal stimulation of tDCS tends to cause varying degrees of skin discomfort, such as tingling, itching, and burning when the current intensity reaches 2 mA, mainly due to mild transient skin effects caused by the vascular expansion effect induced by tES [76]. At the same time, tACS had lower skin discomfort and no significant difference compared to sham stimulation [77]. Due to the activation of the retinal neural firing caused by the placement of the tACS electrode in the frontal and occipital regions, which occurs only during the intervention [78], “optical hallucinations” or “phosphonic responses” are caused. These are perceptions of flickering light without actual visual input. The ipsilateral mandible was suggested as a secondary electrode placement for cerebellar tACS based on findings from a recent study [79] on electrode placement for cerebellar tACS that the cerebellar tACS intervention caused little skin sensation and high-intensity currents in the eye only when the electrodes were positioned close to the frontal and orbital regions, resulting in “optical hallucinations” as a side effect during the tACS intervention. As a result, cerebellar tACS is a painless and secure treatment option. To ensure the safety of the patient’s treatment, the treatment process also necessitates observation, measurement, and questioning of the patient’s blood pressure, heart rate, or any discomfort that may be present. This is followed by a prompt explanation or adjustment of the treatment plan to the patient's tolerance.

### Research Progress on Cerebellar tACS in the Gamma Band

Early in 1995, it was found that a conditional stimulation of 90% AMT given to the contralateral cerebellum 5 ~ 7 ms before M1 stimulation could reduce MEP, and the reduction of MEP was related to the intensity of the conditioned stimulation, by Ugawa Y et al [80, 81]. They discovered that this reduced MEP and that the degree of the MEP reduction correlated with the intensity of the conditioned stimulus. One of the main ways the cerebellum is involved in motor function is through a phenomenon known as cerebellum-brain inhibition (CBI). This phenomenon is thought to be formed by the formation of a dentate-thalamic pathway dominated by Purkinje cells (PC) in the motor areas of the cerebellum for the inhibition of the contralateral motor cortex, which can be used to determine the strength of cerebellar-cortical connections, primarily involving parallel fibers, PC, and Golgi [82]. CBI increased, and MEP decreased after 300-Hz tACS, while CBI decreased, MEP increased, and finger-tapping task time decreased in healthy subjects after 50-Hz true-stimulation tACS, according to research by Naro A et al. [83, 84] using parallel fiber, PC, and Golgi firing frequencies of 10 Hz, 50 Hz, and 300 Hz to control 50-Hz sham-stimulation tACS on the cerebellum, respectively. Only 50 Hz caused an increase in MEP in the ipsilateral lower extremities when the same team applied the same tACS frequency setting to the lower extremities of healthy subjects. Based on this finding, the researchers hypothesized that 50-Hz ctACS might prevent PC-forming cerebellar LTD from increasing MEP 14. Based on the results of the studies mentioned above [35, 36], we can conclude that cerebellar tACS intervention in the 50-Hz gamma band has effects on cerebellar PC that go beyond simple LTD interference. There is insufficient evidence that PC is involved in CBI [85], so CBI is primarily used to explain cerebellar-cortical connections and is not sufficient, although the gamma band of tACS is thought to induce LTD-like plasticity changes [86] in the primary motor circuits of the brain. Additionally, different Purkinje cell synapses have other functions that include facilitation-inhibition and inhibition-inhibition. These functions can selectively inhibit peripheral muscles unrelated to motor production from improving cortical signaling to active muscles during locomotion, making cerebellum activation a convergent enhancement. Therefore, we prefer 50-Hz gamma band cerebellar tACS on walking, which may be connected to the cerebellum’s influence on walking balance, enhanced postural regulation before and during exercise, and the gamma band itself.

It has been shown that [87, 88] tACS in the gamma band of the M1-cerebellum improves visuomotor tracking ability in healthy individuals compared to M1 alone, as well as an increase in the number and speed of muscle fiber recruitment, representing motor ability.

Exogenous gamma oscillations produced by tACS in the primary motor cortex can significantly decrease the abduction reaction time [89] of the nonlethal thumb, whereas the coherence of cerebellar gamma-band oscillations with theta oscillations at 4–8 Hz in the hippocampus itself dominates skill learning and memory retrieval. This indicates that gamma-band changes in the cerebellum not only support the gamma band’s ability to synchronize motor-related brain regions like sensory and motor cortices [90] but also further improve motor performance by enhancing the activation of neural networks related to motor learning, such as vision and memory. This aligns with the earlier assertion that tACS intervention in the cerebellum’s gamma band can activate the cross-regional brain area. Improved upper limb function has been achieved using 50-Hz gamma cerebellar tACS, which uses gamma frequency band entrainment of the cerebellar inhibitory pathway Purkinje cell-parallel fibers. This technique enhances the learning of fixed-sequence movements and subsequently improves the mobility of patterned activities in daily life [91]. The SMA cerebellum was a better target than the supplementary motor area in another trial [92] of gamma-band tACS intervention on the upper extremity in healthy subjects.

Studies on the upper extremity have demonstrated that tACS in the gamma band acting on the cerebellum can support motor relearning in post-stroke patients either through its oscillations or through coherence with oscillations in other frequency bands in adjacent brain regions of the circuit [93], although tACS has received less attention about the use of motor learning theory to improve walking. Therefore, we hypothesize that tACS intervention in the gamma band can have a corresponding effect on gait and posture by activating the motor learning network, and its theory and mechanism are worth further exploring and have some research significance and clinical value. We summarize the current gamma band in motion literature in Table 1.

**Table 1:** Summary of the application of cerebellar gamma oscillations in motion

Author, Year	Sample size (*N*)	Domain	Types of trials and group	Cohort	Stimulation parameters	Duration and course	Assessment content	Outcomes
Kooiman et al.(2020) [61]	14	Re-learn walking; gait pattern	Observational study; none	Healthy participants	None	Not mentioned	Walk on a treadmill without (one block, 4 min) and with a dummy prosthesis (three blocks, 3 × 4 min)	Gait analysis, EEG
Weersink JB et al.(2019) [62]	20	Gait-related arm swing; four-limb co-ordination	Observational study; none	Healthy participants	None	Not mentioned	Walking without and with arm swing; two sessions conducted on the same day with approximately 10 min in between	Gait analysis, EEG
Seeber M et al.(2015) [63]	10	Rhythmicity of walking	Observational study; none	Healthy participants	None	Not mentioned	Four runs (6 min each) of active walking and three runs of 123 upright standing (3 min each) in a robotic gait orthosis	EEG
Naro A et al.(2016) [83]	25	Motor adaptation	Crossover study; none	Healthy participants	10, 50, and 300 Hz and a sham tACS, 2 mA	Immediately, 15 min, and 30 min	Each subject practiced all the conditioning protocols in a random order of stimulation, with a 1-week interval	MEP, CBI, LICI, EEG
Naro A et al.(2017) [84]	15	Motor activity	Crossover study; none	Healthy participants	10, 50, and 300 Hz and a sham tACS, 2 mA	Immediately, 30 min, and 60 min	Each subject practiced all the conditioning protocols in a random order of stimulation, with a 1-week interval	WMFT, MEP, CBI, EEG
Naro A et al.(2017) [14]	25	Gait control	Crossover study; none	Healthy participants	10, 50, and 300 Hz and a sham tACS, 2 mA	Immediately, 20 min, 60 min, and 2 h	Each subject practiced all the conditioning protocols in a random order of stimulation, with a 1-week interval	MEP, CBI, gait analysis
Miyaguchi S et al.(2020) [87]	30	Motor learning	RCT; 2	healthy participants	70 Hz, 1 mA tACS on the right M1 and left cerebellar hemisphere a sham tACS	During tACS, day 2 ~ 5	Not mentioned	EMG, visuomotor control task error rate
Akkad, H et al. (2021) [ 89]	58	Motor learning	RCT; 3	Healthy participants	75 Hz (C4)—6 Hz (Pz), tACS 2 mA; upper panel or lower panel or sham	During tACS	Not mentioned	Behavioral task
Giustiniani, A et al. (2019) [ 91]	17	Motor learning	Crossover study; none	Healthy participants	40 Hz, 1 Hz, or sham tACS. Stimulation intensity was set at 2 mA peak-to-peak, corresponding to 0.08 mA/cm^2^ current density over the M1 hotspot	During tACS	The experiment included three sessions, performed at least 3 days apart, one for each stimulation frequency (40 Hz, 1 Hz, or sham). The sessions order was counterbalanced across subjects	SRTT、MEP

*tACS* transcranial alternating current stimulation, *EEG* electroencephalogram, *MEP* motor evoked potential, *CBI* cerebellum-brain inhibition, *LICI* long-interval intracortical inhibition, *WMFT* Wolf motor function test, *SRTT* serial reaction time task

## Summary and Prospect

The goal of clinical treatment has been to improve postural control, and correct and prevent the occurrence of gait abnormalities so that patients can better reintegrate into their families and society. Rehabilitation of walking and balance after stroke has been the focus of clinical treatment. The cerebellum, which plays a crucial role in balance, postural control, muscle tone, and lower limb coordination in both healthy individuals and stroke patients, has recently gained attention as a second NIBS target after the primary motor cortex due to its relatively intact structural preservation, high plasticity, and these characteristics in patients after episodic stroke. It has been demonstrated that the gamma frequency of tACS is an exogenous frequency band that enhances motor learning. The gamma band is also an important EEG-generating segment of the cerebellum itself at the beginning of the swing phase of the gait cycle and during rhythmic movement changes. The gamma-band cerebellar tACS has been demonstrated to enhance lower limb MEP and upper limb function in healthy people. However, no study has been able to explain how repeated cerebellar tACS at gamma frequency interventions in stroke patients affect the neural circuits linked to abnormal gait, postural control, balance, and motor learning, or what the long-lasting effects are. Is there a particular frequency or band with the best efficacy for movement or motor learning with multiple targets, numerous NIBS approaches, combined interventions with conventional rehabilitation approaches targeting the affected neuromuscular, and various wavelengths in the gamma band? Additionally, the cerebellum’s anatomical makeup and functional mechanisms are intricate, and high-precision cerebellar neuromodulation is an excellent way to explain available mechanisms that merit more research.

## Data Availability

This review paper does not involve any clinical data.
